# 8-[(3,3-Dimethyl­oxiran-2-yl)methoxy­meth­yl]-11-hydr­oxy-2-isopropenyl-5-methyl-12-oxo-1,2,3,12-tetra­hydro­pyrano[3,2-*a*]xanthen-1-yl acetate

**DOI:** 10.1107/S1600536809038343

**Published:** 2009-09-26

**Authors:** Jatupol Liangsakul, Suphongphan Srisurichan, Nongnuj Muangsin, Narongsak Chaichit, Surachai Pornpakakul

**Affiliations:** a Reserch Centre for Bioorganic Chemistry, Department of Chemistry, Faculty of Science, Chulalongkorn University, Phayathai, Bangkok 10330, Thailand; b Department of Physics, Faculty of Science and Technology, Thammasart University, PathumThani 12121, Thailand

## Abstract

The title compound, commonly known as 14-methoxy­tajixanthone-25-acetate, C_28_H_30_O_8_, was isolated from *Emericella variecolor*. The central xanthone core is approximately planar (r.m.s. deviation = 0.084 Å). The dihydro­pyran ring adopts a distorted half-chair conformation. The oxirane plane is oriented at an angle of 63.3 (2)° with respect to the phenol group. An intra­molecular O—H⋯O hydrogen bond forms an *S*(6) ring. In the crystal, mol­ecules are linked into a two-dimensional network parallel to the *ab* plane by weak inter­molecular C—H⋯O hydrogen bonds.

## Related literature

For general background to 14-methoxy­tajixanthone-25-acetate, see: Bringmann *et al.* (2003 ▶); Pornpakakul *et al.* (2006 ▶); Raper & Fennel (1965 ▶). For related structures, see: Fukuyama *et al.* (1978 ▶); Lee *et al.* (2005 ▶).
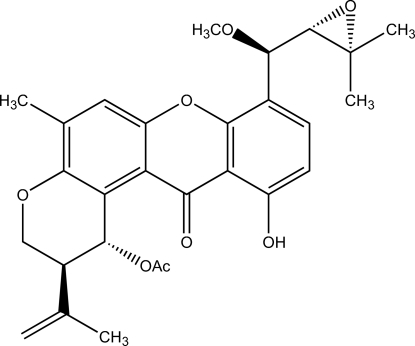


## Experimental

### 

#### Crystal data

C_28_H_30_O_8_
*M* *_r_* = 494.54
Monoclinic, 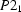

*a* = 11.3323 (1) Å
*b* = 8.8199 (2) Å
*c* = 12.8741 (3) Å
β = 91.765 (1)°
*V* = 1286.15 (4) Å^3^
*Z* = 2
Mo *K*α radiation
μ = 0.09 mm^−1^
*T* = 293 K
0.40 × 0.24 × 0.18 mm

#### Data collection

Bruker SMART area-detector diffractometer
Absorption correction: none
9335 measured reflections
3840 independent reflections
2691 reflections with *I* > 2σ(*I*)
*R*_int_ = 0.030

#### Refinement

*R*[*F* ^2^ > 2σ(*F* ^2^)] = 0.056
*wR*(*F* ^2^) = 0.141
*S* = 1.06
3840 reflections
335 parameters
16 restraints
H-atom parameters constrained
Δρ_max_ = 0.20 e Å^−3^
Δρ_min_ = −0.27 e Å^−3^

### 

Data collection: *SMART* (Bruker, 2006 ▶); cell refinement: *SAINT-Plus* (Bruker, 2006 ▶); data reduction: *SAINT-Plus*; program(s) used to solve structure: *SHELXS97* (Sheldrick, 2008 ▶); program(s) used to refine structure: *SHELXL97* (Sheldrick, 2008 ▶); molecular graphics: *ORTEP-3* (Farrugia, 1997 ▶); software used to prepare material for publication: *publCIF* (Westrip, 2009 ▶).

## Supplementary Material

Crystal structure: contains datablocks global, I. DOI: 10.1107/S1600536809038343/ci2902sup1.cif

Structure factors: contains datablocks I. DOI: 10.1107/S1600536809038343/ci2902Isup2.hkl

Additional supplementary materials:  crystallographic information; 3D view; checkCIF report

## Figures and Tables

**Table 1:** Hydrogen-bond geometry (Å, °)

*D*—H⋯*A*	*D*—H	H⋯*A*	*D*⋯*A*	*D*—H⋯*A*
O4—H4⋯O3	0.82	1.82	2.554 (3)	148
C3—H3*B*⋯O8^i^	0.97	2.57	3.345 (5)	137
C9—H9⋯O4^ii^	0.93	2.55	3.434 (4)	160

Symmetry codes: (i) 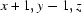; (ii) 
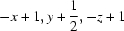
.

**Fig. 1.**
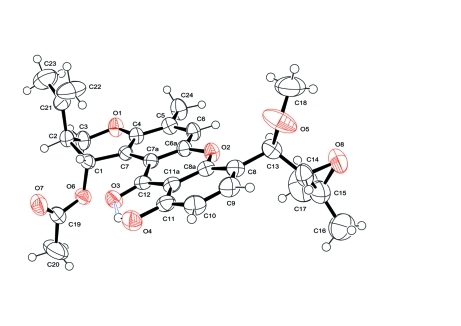
The molecular structure of the title compound. Displacement ellipsoids are drawn at the 30% probability level. The dashed line indicates a hydrogen bond. Only the major disorder component is shown.

**Fig. 2.**
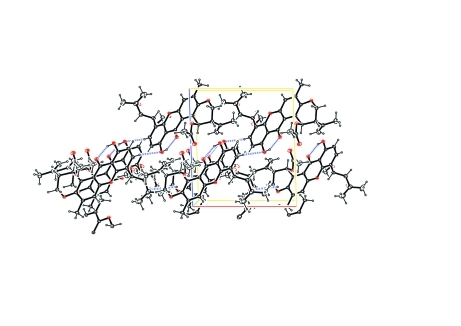
Molecular packing of the title compound, viewed along b-axis. Hydrogen bonds are shown as dashed lines. Only the major disorder component is shown.

## supplementary crystallographic information

### Comment

*Emericella variecolor* is a perfect state of *Aspergillus variecolor*(*syn. Aspergillus stellatus*) (Raper & Fennel, 1965), which produces a variety of compounds such as xanthones (Bringmann *et al.*, 2003; Pornpakakul *et al.*, 2006)

Our research group has investigated metabolites of *Emericella variecolor*, an endophytic fungus of *Croton oblongifolius*. Four xanthones including shamixanthone, 14-methoxytajixanthone-25-acetate (the title compound), tajixanthone methanoate, and tajixanthone hydrate, were isolated from mycelia. All compounds were tested for cytotoxic activity against various human tumor cell lines, including gastric carcinoma, colon carcinoma, breast carcinoma, human hepatocarcinoma, and lung carcinoma. Under the test conditions it was found that 14-methoxy tajixanthone-25-acetate and tajixanthone hydrate are almost as active as doxorubicin hydrochloride against gastric carcinoma (KATO3) and breast carcinoma (BT474) (Pornpakakul *et al.*, 2006). In this work, we report the crystal structure of 14-methoxytajixanthone-25-acetate.

The central xanthone core is approximately planar (r.m.s. deviation of 0.084 Å). The dihydropyran ring adopts a distorted half-chair conformation, with atoms C2 and C3 deviated 0.357 (3)Å and -0.315 (3) Å, respectively, out of the mean plane [r.m.s. deviation 0.214 Å]. The orientation of the isobutene side chain with respect to the acetate substitutent is indicated by the torsion angle O6—C1—C2—C21 of 166.1 (3)°, indicating a anti-periplanar conformation. The oxirane plane is inclined at an angle of 63.3 (2)° with respect to the phenol ring.

The crystal packing is stabilized by weak intermolecular C—H···O interactions (Fig.2).

### Experimental

Seventy-five Erlenmeyer flasks (250 ml) containing malt extract (2 g) and water (100 ml per flask), were autoclaved twice at 121°C for 40 min. Pure culture of *E.variecolor* grown on PDA at room temperature for 7 days were cut into disks 8 mm in a diameter. Two disks were transferred under sterile conditions into each Erlenmeyer flask and then statically incubated for 6 weeks at room temperature. The fermentation broth was filtered through Whatman No.1 filter paper.

The mycelium (253 g wet weight) was extracted with methanol (500 ml × 10) to yield crude  methanol extract that was a dark reddish solid (13.95 g). The dark reddish solid was re-extracted with ethyl acetate (500 ml × 10) to give 5.86 g of crude ethyl acetate extract. This extract was then separated by silica gel column chromatography (230–400 mesh, 100 g) and eluted with an *n*-hexane-ethyl acetate mixture with stepwise increasing polarity. A total of 600 fractions of 100 ml each were collected and combined on the basis of TLC profile. UV light and vanillin/H_2_SO_4_/EtOH reagent were used as detecting methods. The combined fraction (154 mg) contained the title compound as a main component. It was obtained from elution with hexane-EtOAc (80:20) and crystallized to give 14-methoxytajixanthone-25-acetate (75 mg). Suitable single crystals of the title compound were obtained by crystallization from a mixture of benzene/diethyl ether/chloroform.

### Refinement

The methyl group of the methoxy group is disordered over two orientations with occupancies of 0.700 (13) and 0.300 (13). The C—O distances involving the disordered atoms were restrained to be equal and these atoms were subjected to a rigid bond restraint. The displacement parameters of atoms C16 and C17 were restrained to an approximate isotropic behaviour. H atoms were positioned geometrically and refined using a riding model, with C-H = 0.93 Å (aromatic), 0.97 Å (CH_2_), 0.98 Å (CH_3_) and O–H = 0.82 Å, and *U_iso_*(H) = 1.2*U*_eq_(C) and 1.5*U*eq(O and C_methyl_). In the absence of significant anomalous scattering effects, Friedel pairs were averaged.

### Crystal data

**Table:**

C_28_H_30_O_8_	*F*(000) = 524
*M**_r_* = 494.54	*D*_x_ = 1.277 Mg m^−^^3^
Monoclinic, *P*2_1_	Mo *K*α radiation, λ = 0.71073 Å
Hall symbol: P 2yb	Cell parameters from 9335 reflections
*a* = 11.3323 (1) Å	θ = 1.6–30.4°
*b* = 8.8199 (2) Å	µ = 0.09 mm^−^^1^
*c* = 12.8741 (3) Å	*T* = 293 K
β = 91.765 (1)°	Plate, yellow
*V* = 1286.15 (4) Å^3^	0.40 × 0.24 × 0.18 mm
*Z* = 2	

### Data collection

**Table:**

Bruker SMART area-detector diffractometer	2691 reflections with *I* > 2σ(*I*)
Radiation source: fine-focus sealed tube	*R*_int_ = 0.030
graphite	θ_max_ = 30.4°, θ_min_ = 1.6°
ω scans	*h* = −16→15
9335 measured reflections	*k* = −9→12
3840 independent reflections	*l* = −18→17

### Refinement

**Table:**

Refinement on *F*^2^	16 restraints
Least-squares matrix: full	H-atom parameters constrained
*R*[*F*^2^ > 2σ(*F*^2^)] = 0.056	*w* = 1/[σ^2^(*F*_o_^2^) + (0.0608*P*)^2^ + 0.2544*P*] where *P* = (*F*_o_^2^ + 2*F*_c_^2^)/3
*wR*(*F*^2^) = 0.141	(Δ/σ)_max_ = 0.001
*S* = 1.06	Δρ_max_ = 0.20 e Å^−^^3^
3840 reflections	Δρ_min_ = −0.27 e Å^−^^3^
335 parameters	

### Special details

**Table:**

Geometry. All esds (except the esd in the dihedral angle between two l.s. planes) are estimated using the full covariance matrix. The cell esds are taken into account individually in the estimation of esds in distances, angles and torsion angles; correlations between esds in cell parameters are only used when they are defined by crystal symmetry. An approximate (isotropic) treatment of cell esds is used for estimating esds involving l.s. planes.

### Fractional atomic coordinates and isotropic or equivalent isotropic displacement parameters (Å^2^)

**Table:**

	*x*	*y*	*z*	*U*_iso_*/*U*_eq_	Occ. (<1)
O1	1.17752 (18)	0.0959 (3)	0.89869 (16)	0.0466 (6)	
O2	0.77214 (19)	0.4077 (3)	0.79097 (15)	0.0415 (5)	
O3	0.8611 (2)	0.0712 (3)	0.58879 (16)	0.0455 (5)	
O4	0.7032 (2)	0.1668 (3)	0.45891 (18)	0.0542 (7)	
H4	0.7573	0.1133	0.4816	0.081*	
O5	0.6074 (4)	0.7709 (4)	0.6946 (3)	0.0937 (12)	
O6	1.0238 (2)	−0.1231 (3)	0.70433 (16)	0.0444 (5)	
O7	1.0451 (2)	−0.1637 (3)	0.53268 (18)	0.0592 (7)	
O8	0.4404 (3)	0.7222 (4)	0.8668 (3)	0.0780 (9)	
C1	1.0770 (3)	0.0262 (4)	0.6941 (2)	0.0365 (6)	
H1	1.0648	0.0638	0.6230	0.044*	
C2	1.2093 (3)	0.0120 (4)	0.7207 (2)	0.0435 (7)	
H2	1.2384	−0.0757	0.6823	0.052*	
C3	1.2211 (3)	−0.0264 (5)	0.8359 (2)	0.0483 (8)	
H3A	1.1768	−0.1180	0.8496	0.058*	
H3B	1.3033	−0.0454	0.8544	0.058*	
C4	1.0734 (3)	0.1616 (4)	0.8659 (2)	0.0380 (7)	
C5	1.0267 (3)	0.2659 (4)	0.9370 (2)	0.0412 (7)	
C6	0.9258 (3)	0.3443 (4)	0.9087 (2)	0.0420 (7)	
H6	0.8946	0.4148	0.9540	0.050*	
C6A	0.8702 (3)	0.3185 (4)	0.8119 (2)	0.0374 (6)	
C7	1.0181 (2)	0.1313 (4)	0.7703 (2)	0.0336 (6)	
C7A	0.9106 (3)	0.2103 (4)	0.7430 (2)	0.0341 (6)	
C8	0.6238 (3)	0.5019 (4)	0.6755 (2)	0.0415 (7)	
C8A	0.7130 (3)	0.3972 (4)	0.6967 (2)	0.0374 (7)	
C9	0.5634 (3)	0.4873 (5)	0.5797 (3)	0.0490 (8)	
H9	0.5037	0.5563	0.5632	0.059*	
C10	0.5881 (3)	0.3753 (5)	0.5083 (3)	0.0502 (9)	
H10	0.5442	0.3683	0.4463	0.060*	
C11	0.6781 (3)	0.2743 (4)	0.5299 (2)	0.0424 (7)	
C11A	0.7432 (3)	0.2835 (4)	0.6255 (2)	0.0370 (6)	
C12	0.8409 (3)	0.1797 (4)	0.6476 (2)	0.0364 (7)	
C13	0.5943 (3)	0.6298 (4)	0.7483 (3)	0.0468 (8)	
H13	0.6479	0.6272	0.8094	0.056*	
C14	0.4695 (3)	0.6246 (5)	0.7819 (3)	0.0576 (10)	
H14	0.4104	0.6229	0.7246	0.069*	
C15	0.4291 (5)	0.5607 (6)	0.8791 (4)	0.0771 (13)	
C16	0.3022 (6)	0.5108 (11)	0.8848 (6)	0.133 (3)	
H16A	0.2725	0.5372	0.9515	0.200*	
H16B	0.2974	0.4030	0.8754	0.200*	
H16C	0.2558	0.5604	0.8312	0.200*	
C17	0.5144 (7)	0.4907 (11)	0.9560 (5)	0.132 (3)	
H17A	0.4787	0.4841	1.0225	0.197*	
H17B	0.5843	0.5521	0.9618	0.197*	
H17C	0.5351	0.3909	0.9330	0.197*	
C18	0.6673 (8)	0.8824 (9)	0.7449 (9)	0.098 (4)	0.694 (16)
H18A	0.6714	0.9699	0.7007	0.147*	0.694 (16)
H18B	0.7458	0.8481	0.7626	0.147*	0.694 (16)
H18C	0.6275	0.9087	0.8071	0.147*	0.694 (16)
C18'	0.7012 (12)	0.841 (2)	0.6681 (17)	0.082 (6)	0.306 (16)
H18D	0.7277	0.8013	0.6036	0.123*	0.306 (16)
H18E	0.7619	0.8284	0.7211	0.123*	0.306 (16)
H18F	0.6840	0.9475	0.6600	0.123*	0.306 (16)
C19	1.0135 (3)	−0.2066 (4)	0.6160 (3)	0.0502 (8)	
C20	0.9601 (6)	−0.3577 (6)	0.6382 (4)	0.1004 (19)	
H20A	0.9764	−0.3843	0.7095	0.151*	
H20B	0.9934	−0.4329	0.5938	0.151*	
H20C	0.8763	−0.3531	0.6255	0.151*	
C21	1.2826 (3)	0.1480 (5)	0.6893 (3)	0.0517 (9)	
C22	1.2410 (4)	0.2625 (6)	0.6336 (4)	0.0888 (16)	
H22A	1.2908	0.3415	0.6157	0.107*	
H22B	1.1619	0.2644	0.6122	0.107*	
C23	1.4093 (4)	0.1446 (8)	0.7237 (4)	0.0872 (15)	
H23A	1.4408	0.0450	0.7128	0.131*	
H23B	1.4161	0.1695	0.7963	0.131*	
H23C	1.4526	0.2171	0.6844	0.131*	
C24	1.0876 (3)	0.2921 (5)	1.0420 (2)	0.0574 (10)	
H24A	1.1662	0.3290	1.0322	0.086*	
H24B	1.0913	0.1984	1.0799	0.086*	
H24C	1.0440	0.3655	1.0803	0.086*	

### Atomic displacement parameters (Å^2^)

**Table:**

	*U*^11^	*U*^22^	*U*^33^	*U*^12^	*U*^13^	*U*^23^
O1	0.0437 (12)	0.0556 (15)	0.0402 (11)	0.0145 (11)	−0.0051 (9)	−0.0011 (10)
O2	0.0442 (12)	0.0433 (13)	0.0365 (10)	0.0112 (10)	−0.0044 (9)	−0.0029 (9)
O3	0.0512 (12)	0.0423 (13)	0.0429 (11)	0.0013 (11)	−0.0031 (9)	−0.0102 (10)
O4	0.0515 (13)	0.0636 (17)	0.0469 (12)	−0.0014 (13)	−0.0099 (10)	−0.0169 (12)
O5	0.163 (4)	0.0434 (17)	0.0730 (19)	−0.019 (2)	−0.031 (2)	0.0128 (15)
O6	0.0558 (13)	0.0356 (12)	0.0424 (11)	−0.0041 (11)	0.0086 (9)	−0.0017 (10)
O7	0.0778 (17)	0.0538 (16)	0.0467 (13)	0.0035 (15)	0.0126 (12)	−0.0099 (12)
O8	0.081 (2)	0.0619 (19)	0.091 (2)	0.0141 (17)	0.0095 (17)	−0.0240 (17)
C1	0.0421 (16)	0.0315 (15)	0.0361 (14)	−0.0008 (13)	0.0043 (12)	−0.0003 (12)
C2	0.0423 (16)	0.0416 (18)	0.0471 (16)	0.0062 (15)	0.0071 (13)	−0.0046 (14)
C3	0.0473 (18)	0.051 (2)	0.0468 (17)	0.0155 (17)	0.0012 (14)	0.0001 (16)
C4	0.0396 (15)	0.0394 (17)	0.0348 (14)	0.0059 (14)	−0.0006 (11)	0.0017 (13)
C5	0.0469 (17)	0.0446 (18)	0.0318 (14)	0.0031 (15)	−0.0032 (12)	0.0016 (13)
C6	0.0477 (17)	0.0451 (18)	0.0331 (14)	0.0090 (15)	−0.0003 (12)	−0.0062 (13)
C6A	0.0373 (15)	0.0379 (16)	0.0370 (14)	0.0037 (13)	0.0001 (11)	0.0001 (13)
C7	0.0357 (14)	0.0329 (15)	0.0322 (13)	−0.0003 (13)	0.0020 (11)	0.0001 (12)
C7A	0.0363 (14)	0.0353 (15)	0.0306 (13)	−0.0024 (13)	0.0019 (11)	−0.0001 (11)
C8	0.0411 (16)	0.0392 (18)	0.0439 (16)	−0.0010 (15)	−0.0031 (13)	0.0029 (14)
C8A	0.0343 (15)	0.0400 (16)	0.0378 (15)	−0.0039 (14)	−0.0007 (12)	0.0030 (13)
C9	0.0422 (17)	0.052 (2)	0.0516 (18)	0.0034 (17)	−0.0114 (14)	0.0077 (17)
C10	0.0459 (17)	0.061 (2)	0.0430 (16)	−0.0015 (18)	−0.0148 (13)	−0.0003 (16)
C11	0.0354 (15)	0.052 (2)	0.0400 (15)	−0.0087 (15)	−0.0015 (12)	−0.0021 (15)
C11A	0.0337 (14)	0.0429 (17)	0.0342 (14)	−0.0046 (14)	−0.0007 (11)	0.0027 (13)
C12	0.0361 (14)	0.0367 (17)	0.0363 (14)	−0.0090 (13)	0.0008 (12)	0.0013 (13)
C13	0.0542 (19)	0.0408 (18)	0.0447 (17)	0.0050 (16)	−0.0106 (14)	0.0046 (15)
C14	0.057 (2)	0.056 (2)	0.060 (2)	0.0177 (19)	−0.0058 (16)	−0.0091 (18)
C15	0.085 (3)	0.069 (3)	0.079 (3)	−0.004 (3)	0.024 (2)	−0.012 (2)
C16	0.117 (4)	0.136 (6)	0.151 (5)	−0.031 (4)	0.065 (4)	−0.048 (5)
C17	0.169 (6)	0.134 (6)	0.093 (4)	0.025 (5)	0.026 (4)	0.042 (4)
C18	0.087 (6)	0.063 (5)	0.144 (9)	−0.021 (4)	−0.005 (6)	0.013 (5)
C18'	0.095 (11)	0.060 (9)	0.093 (13)	0.026 (8)	0.020 (9)	−0.002 (9)
C19	0.058 (2)	0.0392 (19)	0.0541 (19)	−0.0021 (17)	0.0097 (16)	−0.0102 (16)
C20	0.152 (5)	0.057 (3)	0.094 (3)	−0.043 (3)	0.033 (3)	−0.022 (3)
C21	0.0450 (18)	0.057 (2)	0.0532 (19)	−0.0070 (18)	0.0084 (14)	−0.0058 (18)
C22	0.073 (3)	0.074 (3)	0.119 (4)	−0.026 (3)	−0.003 (3)	0.035 (3)
C23	0.060 (2)	0.109 (4)	0.093 (3)	−0.022 (3)	−0.001 (2)	0.000 (3)
C24	0.064 (2)	0.070 (3)	0.0380 (16)	0.017 (2)	−0.0119 (15)	−0.0120 (17)

### Geometric parameters (Å, °)

**Table:**

O1—C4	1.370 (4)	C9—H9	0.93
O1—C3	1.444 (4)	C10—C11	1.376 (5)
O2—C8A	1.372 (4)	C10—H10	0.93
O2—C6A	1.381 (4)	C11—C11A	1.418 (4)
O3—C12	1.246 (4)	C11A—C12	1.458 (4)
O4—C11	1.353 (4)	C13—C14	1.492 (5)
O4—H4	0.82	C13—H13	0.98
O5—C18'	1.287 (11)	C14—C15	1.459 (6)
O5—C18	1.350 (8)	C14—H14	0.98
O5—C13	1.434 (5)	C15—C17	1.496 (9)
O6—C19	1.357 (4)	C15—C16	1.508 (8)
O6—C1	1.456 (4)	C16—H16A	0.96
O7—C19	1.202 (4)	C16—H16B	0.96
O8—C14	1.438 (5)	C16—H16C	0.96
O8—C15	1.439 (6)	C17—H17A	0.96
C1—C7	1.519 (4)	C17—H17B	0.96
C1—C2	1.533 (4)	C17—H17C	0.96
C1—H1	0.98	C18—H18A	0.96
C2—C21	1.520 (5)	C18—H18B	0.96
C2—C3	1.523 (4)	C18—H18C	0.96
C2—H2	0.98	C18'—H18D	0.96
C3—H3A	0.97	C18'—H18E	0.96
C3—H3B	0.97	C18'—H18F	0.96
C4—C7	1.390 (4)	C19—C20	1.495 (6)
C4—C5	1.412 (4)	C20—H20A	0.96
C5—C6	1.376 (4)	C20—H20B	0.96
C5—C24	1.516 (4)	C20—H20C	0.96
C6—C6A	1.398 (4)	C21—C22	1.318 (6)
C6—H6	0.93	C21—C23	1.490 (5)
C6A—C7A	1.389 (4)	C22—H22A	0.93
C7—C7A	1.437 (4)	C22—H22B	0.93
C7A—C12	1.465 (4)	C23—H23A	0.96
C8—C8A	1.390 (5)	C23—H23B	0.96
C8—C9	1.397 (4)	C23—H23C	0.96
C8—C13	1.511 (5)	C24—H24A	0.96
C8A—C11A	1.408 (4)	C24—H24B	0.96
C9—C10	1.384 (5)	C24—H24C	0.96
			
C4—O1—C3	116.7 (2)	C14—C13—C8	112.8 (3)
C8A—O2—C6A	120.1 (2)	O5—C13—H13	109.7
C11—O4—H4	109.5	C14—C13—H13	109.7
C18'—O5—C18	50.7 (9)	C8—C13—H13	109.7
C18'—O5—C13	130.2 (9)	O8—C14—C15	59.5 (3)
C18—O5—C13	117.3 (5)	O8—C14—C13	116.3 (3)
C19—O6—C1	116.1 (2)	C15—C14—C13	125.9 (4)
C14—O8—C15	61.0 (3)	O8—C14—H14	114.5
O6—C1—C7	107.6 (2)	C15—C14—H14	114.5
O6—C1—C2	108.1 (3)	C13—C14—H14	114.5
C7—C1—C2	110.6 (2)	O8—C15—C14	59.5 (3)
O6—C1—H1	110.1	O8—C15—C17	115.1 (6)
C7—C1—H1	110.1	C14—C15—C17	120.9 (5)
C2—C1—H1	110.1	O8—C15—C16	112.5 (5)
C21—C2—C3	113.7 (3)	C14—C15—C16	118.6 (5)
C21—C2—C1	114.5 (3)	C17—C15—C16	116.5 (6)
C3—C2—C1	106.9 (2)	C15—C16—H16A	109.5
C21—C2—H2	107.1	C15—C16—H16B	109.5
C3—C2—H2	107.1	H16A—C16—H16B	109.5
C1—C2—H2	107.1	C15—C16—H16C	109.5
O1—C3—C2	111.0 (3)	H16A—C16—H16C	109.5
O1—C3—H3A	109.4	H16B—C16—H16C	109.5
C2—C3—H3A	109.4	C15—C17—H17A	109.5
O1—C3—H3B	109.4	C15—C17—H17B	109.5
C2—C3—H3B	109.4	H17A—C17—H17B	109.5
H3A—C3—H3B	108.0	C15—C17—H17C	109.5
O1—C4—C7	123.5 (3)	H17A—C17—H17C	109.5
O1—C4—C5	114.3 (2)	H17B—C17—H17C	109.5
C7—C4—C5	122.2 (3)	O5—C18—H18A	109.5
C6—C5—C4	118.7 (3)	O5—C18—H18B	109.5
C6—C5—C24	120.8 (3)	H18A—C18—H18B	109.5
C4—C5—C24	120.5 (3)	O5—C18—H18C	109.5
C5—C6—C6A	120.2 (3)	H18A—C18—H18C	109.5
C5—C6—H6	119.9	H18B—C18—H18C	109.5
C6A—C6—H6	119.9	O5—C18'—H18D	109.5
O2—C6A—C7A	123.1 (3)	O5—C18'—H18E	109.5
O2—C6A—C6	114.7 (3)	H18D—C18'—H18E	109.5
C7A—C6A—C6	122.1 (3)	O5—C18'—H18F	109.5
C4—C7—C7A	118.6 (3)	H18D—C18'—H18F	109.5
C4—C7—C1	119.6 (3)	H18E—C18'—H18F	109.5
C7A—C7—C1	121.6 (2)	O7—C19—O6	123.8 (3)
C6A—C7A—C7	118.1 (3)	O7—C19—C20	125.7 (4)
C6A—C7A—C12	118.7 (3)	O6—C19—C20	110.4 (3)
C7—C7A—C12	123.1 (3)	C19—C20—H20A	109.5
C8A—C8—C9	116.4 (3)	C19—C20—H20B	109.5
C8A—C8—C13	123.3 (3)	H20A—C20—H20B	109.5
C9—C8—C13	120.3 (3)	C19—C20—H20C	109.5
O2—C8A—C8	117.5 (3)	H20A—C20—H20C	109.5
O2—C8A—C11A	120.2 (3)	H20B—C20—H20C	109.5
C8—C8A—C11A	122.4 (3)	C22—C21—C23	120.2 (4)
C10—C9—C8	123.3 (3)	C22—C21—C2	124.1 (3)
C10—C9—H9	118.4	C23—C21—C2	115.7 (4)
C8—C9—H9	118.4	C21—C22—H22A	120.0
C11—C10—C9	119.5 (3)	C21—C22—H22B	120.0
C11—C10—H10	120.3	H22A—C22—H22B	120.0
C9—C10—H10	120.3	C21—C23—H23A	109.5
O4—C11—C10	119.2 (3)	C21—C23—H23B	109.5
O4—C11—C11A	120.8 (3)	H23A—C23—H23B	109.5
C10—C11—C11A	120.0 (3)	C21—C23—H23C	109.5
C8A—C11A—C11	118.4 (3)	H23A—C23—H23C	109.5
C8A—C11A—C12	121.2 (2)	H23B—C23—H23C	109.5
C11—C11A—C12	120.4 (3)	C5—C24—H24A	109.5
O3—C12—C11A	121.1 (3)	C5—C24—H24B	109.5
O3—C12—C7A	123.1 (3)	H24A—C24—H24B	109.5
C11A—C12—C7A	115.8 (3)	C5—C24—H24C	109.5
O5—C13—C14	106.2 (3)	H24A—C24—H24C	109.5
O5—C13—C8	108.7 (3)	H24B—C24—H24C	109.5
			
C19—O6—C1—C7	146.2 (3)	C8—C9—C10—C11	−1.7 (6)
C19—O6—C1—C2	−94.3 (3)	C9—C10—C11—O4	−178.8 (3)
O6—C1—C2—C21	166.1 (3)	C9—C10—C11—C11A	1.3 (5)
C7—C1—C2—C21	−76.3 (3)	O2—C8A—C11A—C11	178.6 (3)
O6—C1—C2—C3	−67.0 (3)	C8—C8A—C11A—C11	−1.5 (4)
C7—C1—C2—C3	50.6 (3)	O2—C8A—C11A—C12	−3.0 (4)
C4—O1—C3—C2	43.6 (4)	C8—C8A—C11A—C12	177.0 (3)
C21—C2—C3—O1	63.2 (4)	O4—C11—C11A—C8A	−179.7 (3)
C1—C2—C3—O1	−64.2 (4)	C10—C11—C11A—C8A	0.2 (5)
C3—O1—C4—C7	−9.1 (4)	O4—C11—C11A—C12	1.8 (4)
C3—O1—C4—C5	171.8 (3)	C10—C11—C11A—C12	−178.3 (3)
O1—C4—C5—C6	176.3 (3)	C8A—C11A—C12—O3	173.7 (3)
C7—C4—C5—C6	−2.8 (5)	C11—C11A—C12—O3	−7.9 (4)
O1—C4—C5—C24	−2.9 (5)	C8A—C11A—C12—C7A	−5.3 (4)
C7—C4—C5—C24	178.0 (3)	C11—C11A—C12—C7A	173.2 (3)
C4—C5—C6—C6A	1.1 (5)	C6A—C7A—C12—O3	−169.7 (3)
C24—C5—C6—C6A	−179.7 (3)	C7—C7A—C12—O3	8.9 (5)
C8A—O2—C6A—C7A	−3.2 (4)	C6A—C7A—C12—C11A	9.2 (4)
C8A—O2—C6A—C6	177.3 (3)	C7—C7A—C12—C11A	−172.2 (3)
C5—C6—C6A—O2	−177.9 (3)	C18'—O5—C13—C14	165.3 (14)
C5—C6—C6A—C7A	2.6 (5)	C18—O5—C13—C14	105.2 (7)
O1—C4—C7—C7A	−178.3 (3)	C18'—O5—C13—C8	−73.1 (14)
C5—C4—C7—C7A	0.7 (5)	C18—O5—C13—C8	−133.2 (6)
O1—C4—C7—C1	−3.2 (5)	C8A—C8—C13—O5	121.2 (4)
C5—C4—C7—C1	175.8 (3)	C9—C8—C13—O5	−56.5 (4)
O6—C1—C7—C4	98.4 (3)	C8A—C8—C13—C14	−121.2 (3)
C2—C1—C7—C4	−19.5 (4)	C9—C8—C13—C14	61.1 (4)
O6—C1—C7—C7A	−86.6 (3)	C15—O8—C14—C13	−117.9 (4)
C2—C1—C7—C7A	155.5 (3)	O5—C13—C14—O8	−73.3 (4)
O2—C6A—C7A—C7	175.9 (3)	C8—C13—C14—O8	167.7 (3)
C6—C6A—C7A—C7	−4.6 (4)	O5—C13—C14—C15	−143.4 (4)
O2—C6A—C7A—C12	−5.4 (4)	C8—C13—C14—C15	97.6 (5)
C6—C6A—C7A—C12	174.1 (3)	C14—O8—C15—C17	112.5 (5)
C4—C7—C7A—C6A	2.9 (4)	C14—O8—C15—C16	−111.0 (5)
C1—C7—C7A—C6A	−172.1 (3)	C13—C14—C15—O8	102.1 (5)
C4—C7—C7A—C12	−175.7 (3)	O8—C14—C15—C17	−102.7 (6)
C1—C7—C7A—C12	9.3 (4)	C13—C14—C15—C17	−0.6 (8)
C6A—O2—C8A—C8	−172.6 (3)	O8—C14—C15—C16	100.6 (6)
C6A—O2—C8A—C11A	7.4 (4)	C13—C14—C15—C16	−157.3 (5)
C9—C8—C8A—O2	−178.9 (3)	C1—O6—C19—O7	−0.2 (5)
C13—C8—C8A—O2	3.3 (5)	C1—O6—C19—C20	178.8 (4)
C9—C8—C8A—C11A	1.1 (5)	C3—C2—C21—C22	−130.7 (4)
C13—C8—C8A—C11A	−176.6 (3)	C1—C2—C21—C22	−7.3 (6)
C8A—C8—C9—C10	0.4 (5)	C3—C2—C21—C23	50.6 (4)
C13—C8—C9—C10	178.3 (3)	C1—C2—C21—C23	173.9 (3)

### Hydrogen-bond geometry (Å, °)

**Table:**

*D*—H···*A*	*D*—H	H···*A*	*D*···*A*	*D*—H···*A*
O4—H4···O3	0.82	1.82	2.554 (3)	148
C3—H3B···O8^i^	0.97	2.57	3.345 (5)	137
C9—H9···O4^ii^	0.93	2.55	3.434 (4)	160

Symmetry codes: (i) *x*+1, *y*−1, *z*; (ii) −*x*+1, *y*+1/2, −*z*+1.
